# Association between Osteoprotegerin rs2073618 polymorphism and peri-implantitis susceptibility: a meta-analysis

**DOI:** 10.1186/s12903-022-02657-6

**Published:** 2022-12-12

**Authors:** Min Xu, Churen Zhang, Ye Han, Zhaoguo Yue, Chang Shu, Jianxia Hou

**Affiliations:** 1 Department of Periodontology, National Engineering Laboratory for Digital and Material Technology of Stomatology, Beijing Key Laboratory of Digital Stomatology, Peking University School and Hospital of Stomatology, No. 22 ZhongGuanCun Nandajie, HaiDian District, Beijing, 100081 China; 2 Department of Stomatology, School of Medicine, The First Affiliated Hospital of Xiamen University, Xiamen University, Xiamen, China

**Keywords:** Peri-implantitis, Osteoprotegerin, Meta-analysis, Single nucleotide polymorphism

## Abstract

**Objectives:**

Peri-implantitis was an inflammatory progress on the tissue around the implant. The Osteoprotegerin G1181C (rs2073618) polymorphism was reported to be related to the increased risk of the peri-implantitis, whereas another found no relationship. The present study was conducted to research the relationship between Osteoprotegerin rs2073618 polymorphism and peri-implantitis susceptibility.

**Materials and methods:**

The meta-analysis was performed according to the Preferred Reporting Items for Systematic reviews. Electronic databases including PubMed, Web of science, Springer Link and Embase (updated to April 15, 2022) were retrieved. The cohort study, case-control study or cross-sectional study focusing on the Osteoprotegerin rs2073618 polymorphism and peri-implantitis were retrieved. The data included basic information of each study and the genotype and allele frequencies of the cases and controls.

**Results:**

Three studies were finally included, including 160 cases and 271 controls. Allelic model, homozygote model, recessive model, dominant model, and heterozygous model were established to assess the relationship between OPG rs2073618 polymorphism and peri-implantitis susceptibility. The Osteoprotegerin rs2073618 polymorphism was significantly associated with peri-implantitis in Recessive model and Homozygote model.

**Conclusion:**

OPG rs2073618 polymorphism in Recessive model and Homozygote model was highly likely related to the risk of peri-implantitis.

*PROSPERO registration number*: CRD42022320812

**Supplementary Information:**

The online version contains supplementary material available at 10.1186/s12903-022-02657-6.

## Introduction

### Background

With the development and maturity of implant technology, dental implants have become the main means of restoring missing teeth, in the train of which follows is the appearance of peri-implantitis [1]. Peri-implantitis showed clinical symptoms similar to those of periodontitis, characterized by redness, bleeding of the soft tissue, and loss of bone tissue around the implant [2]. Different studies have reported that the incidence of peri-implantitis varies, ranging from 7 to 56% [3–5]. The occurrence of peri-implantitis is affected by many factors, including history of periodontitis, plaque microorganisms, smoking, implant design and genetic factors [6]. It has been demonstrated that patients who have experienced implantation failure have a higher risk of failure again, and this phenomenon may be related to genetic polymorphism [7]. Therefore, genetic susceptibility is an important part of the research on the etiology of peri-implant inflammation.

Bone resorption is a complex mechanism involving the release of several cytokines and apoptosis. Osteoprotegerin (OPG) is closely related to bone resorption process, which is involved in the regulation of osteoclast formation, and belongs to the tumor necrosis factor (TNF) receptor superfamily, which is also known as osteoclastogenesis inhibitory factor (OCIF) [8]. As a receptor of nuclear factor κB receptor activator ligand (RANKL), OPG can restrain the differentiation and activation of osteoclast [9]. It has been reported that the level of OPG in gingival crevicular fluid decreased in patients with severe periodontitis, suggesting that OPG was involved in alveolar bone destruction [10].

### Objective

Previous findings are inconsistent. Therefore, a meta-analysis evaluating the association between OPG rs2073618 polymorphism and peri-implantitis susceptibility was performed in this study. Through the screening of published works, the information about OPG rs2073618 polymorphism from patients with peri-implantitis was gathered for collecting analysis. Do adults with OPG rs2073618 polymorphism, compared to those do not have OPG rs2073618 polymorphism, have higher prevalence of peri-implantitis? To our knowledge, this is the first meta-analysis focusing the relationship between OPG rs2073618 polymorphism and peri-implantitis susceptibility.

## Material and methods

### Information sources and search strategy

The meta-analysis was performed on the basis of the Preferred Reporting Items for Systematic reviews [14]. The current meta-analysis was registered as a priori program in PROSPERO, with the registration number CRD42022320812. Electronic databases including PubMed, Web of science, Springer Link and Embase (updated to April 15, 2022) were retrieved. The search terms were listed below: (“single nucleotide polymorphism” or “SNP”) and “peri-implantitis” and (“osteoprotegerin” or “osteoclastogenesis inhibitory factor” or “OPG” or “OCIF”). In addition, volumes of *Journal of Clinical Periodontology, Journal of Periodontology, Clinical Oral Implants Research* published in 2021 were manually searched.

### Selection process

Two reviewers (M.X and CR.Z) independently screened the literature. Preliminary screening was conducted by reading the title and abstract to sort the articles adhering to the inclusion standards. Then the second screening was carried out by reading the full text of the literature. If the two reviewers held different opinions, another investigator (JX.H) would join the discussion to make a decision. The selected studies would be double-checked by another investigator, to ensure the integrity of the results.

### Eligibility criteria

Eligible studies should meet the following criteria: (1) Types of studies: cohort study, case-control study or cross-sectional study; (2) Patients: subjects with peri-implantitis, diagnosed according to the criteria including peri-implant probing depth more than 5 mm, bleeding on probing, progressive crestal bone loss; (3) Index: allelic variants at OPG rs2073618; (4) Control: systemically healthy subjects with healthy implants and no periodontitis history; (5) Outcome: peri-implantitis susceptibility assessed by odds ratio; (6) Studies published in English; (7) Studies providing enough data, including genotypes and simple size etc. Animal experiments, abstracts and reviews were excluded.

### Quality assessment

Newcastle-Ottawa scale (NOS) [15] was used to evaluate the quality of the included studies about the case definition, comparability of cases and controls and ascertainment of exposure etc. The quality assessment was conducted independently by two reviewers (M.X and CR.Z). If the two reviewers held different opinions, another investigator (JX.H) would join the discussion to make a decision.

### Data collection process

The data extraction was carried out independently by two reviewers (M.X and CR.Z). Another investigator (JX.H) would finally check the extracted data. The content of data extraction included: (1) basic information of the study (the first author, publication time); (2) study race; (3) characteristics of the participants (number, age, gender); (4) Hardy-Weinberg equilibrium (HWE) for the healthy control; (5) genotype, allele frequencies of the cases and controls.

### Effect measures

For the eligible studies, Pearson’s chi-square test was applied to assess the HWE of genotype of the controls. Odds ratio and 95% confidence interval were calculated using Allelic model (C versus G), Heterozygote model (GC versus GG), Recessive model (CC versus GC + GG), Dominant model (GC + CC versus GG), and Homozygote model (CC versus GG).

### Study risk of bias assessment

Heterogeneity between studies was assessed using I^2^ statistics [16]. When P < 0.05 or I^2^ > 50%, assuming a high degree of heterogeneity between the studies, therefore a random effects model was used. Otherwise, the fixed effects model (Mantel-Haenszel method) was used for analysis. Begg’s was adopted to evaluate the publication bias. Data analyzing and processing were conducted by STATA version 12.0 (STATA Corp, College Station, TX, USA).

### Patient and public involvement

None.

## Results

### Basic information of the eligible studies

**Fig. 1:**
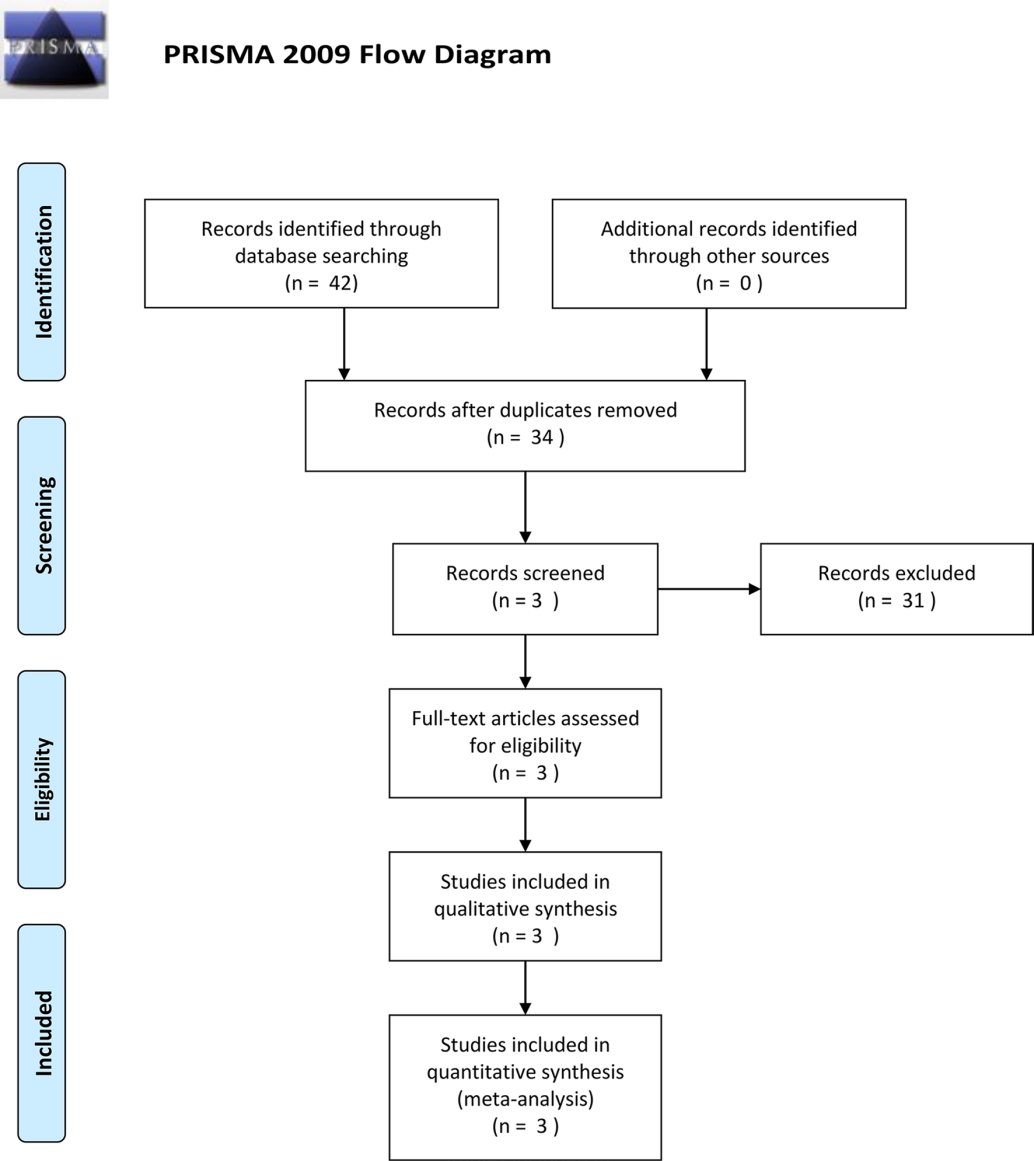
The flowchart of literature retrieve

There were 42 studies identified in the electronic databases. None eligible study was found in other sources. After eliminating the repeated researches, irrelevant studies and reviews, three researches [11, 13, 17] were finally incorporated into this meta-analysis, consisting of 160 cases and 271 controls. The searching and retrieving progress were showed in Fig. 1. NOS scores of eligible studies ranged from 7 to 8, representing the high quality [18]. Data extracted from the included studies was showed in Table 1 (detailed workflow in Additional file 1: Table S1).

**Table 1:** Basic information of eligible studies

Study ID	Year	Country	Ethnicity	Genotyping method	Case			Control			P for HWE∗	NOS
					GG	GC	CC	GG	GC	CC		
Kadkhodazadeh et al.	2012	Iran	Iranian	PCR-RFLP	31	4	5	55	30	4	0.972	7
Silva et al.	2020	Brazil	Brazilian	RT-qPCR	2	2	6	14	30	22	0.53	8
Zhou et al.	2016	China	Chinese	PCR-RFLP	31	50	29	42	56	18	0.925	7

The general information of included studies, containing first author, publication year, country, ethnicity, genotyping methods and number of each genotype. Abbreviations: HWE, Hardy-Weinberg equilibrium; NOS, Newcastle-Ottawa scale.

### Meta-analysis results

**Fig. 2:**
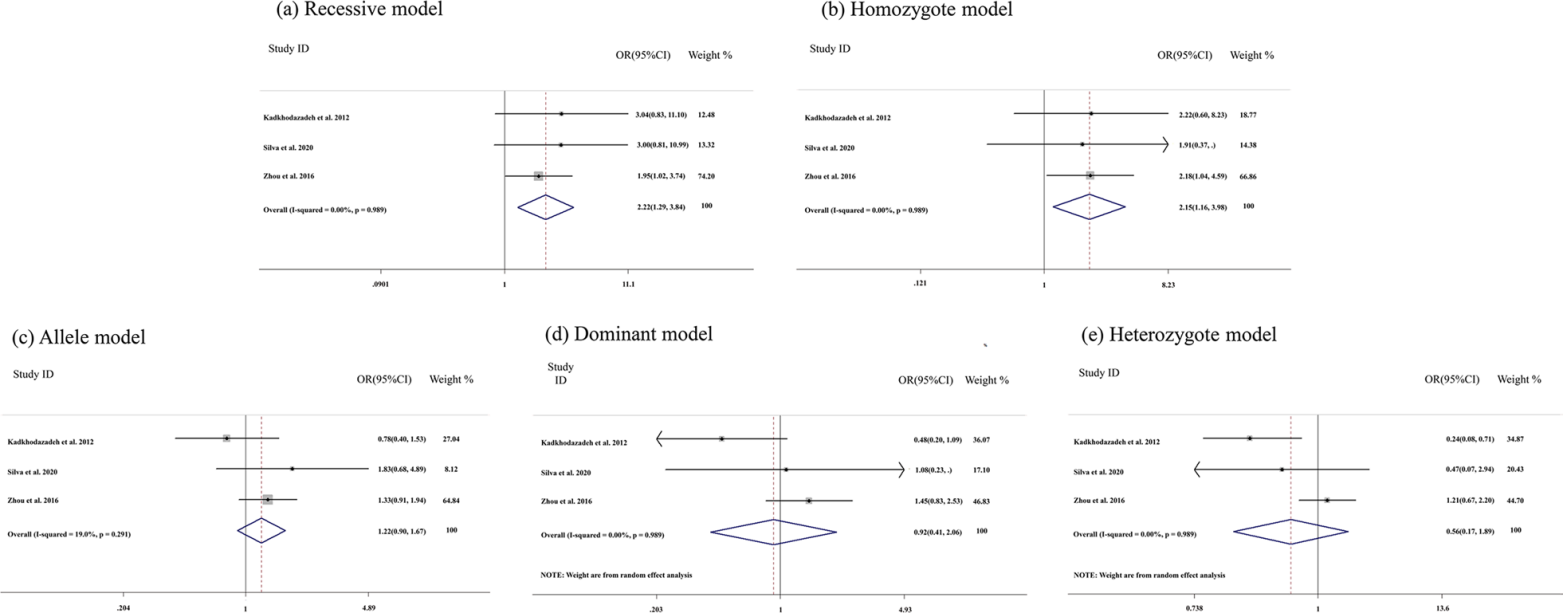
Forest figure for peri-implantitis and OPG rs2073618 polymorphism in different gene models. **a** Recessive model; **b** Homozygote model; **c** Allele model; **d** Dominant model;**e** Heterozygote model

Three studies were adopted to assess the association between the OPG rs2073618 polymorphism and peri-implantitis. The OPG rs2073618 polymorphism was significantly associated with peri-implantitis in Recessive model (CC vs. GG + GC: OR = 2.22, 95% CI: 1.29–3.84, *P* = 0.765 for heterogeneity) and Homozygote model (CC vs. GG: OR = 2.15, 95% CI: 1.16–3.98, *P* = 0.989 for heterogeneity), not in Allele model, Dominant model and Heterozygote model. The results were showed in Fig. 2; Table 2.

**Table 2:** Results of this meta-analysis about OPG rs2073618 polymorphism and peri-implantitis

Polymorphisms	Test of association		Test of heterogeneity		I^2^	Publication bias
	OR (95%)	*P*	Model	*P*		
CC vs GG + GC	2.22 (1.29–3.84)	0.003	Fixed effect	0.765	0	0.12
CC vs GG	2.15 (1.16–3.98)	0.014	Fixed effect	0.989	0	0.12
C vs G	1.22 (0.90, 1.67)	0.206	Fixed effect	0.291	19	0.60
GC + CC vs GG	0.92 (0.41, 2.06)	0.28	Random effecf	0.098	56.9	0.60
GC vs GG	0.56 (0.17, 1.89)	0.353	Random effecf	0.037	69.7	0.60

Odds ratio and heterogeneity of each gene model. The *P* value of Begg’s test was listed below the Publication bias

### Publication bias

**Fig. 3:**
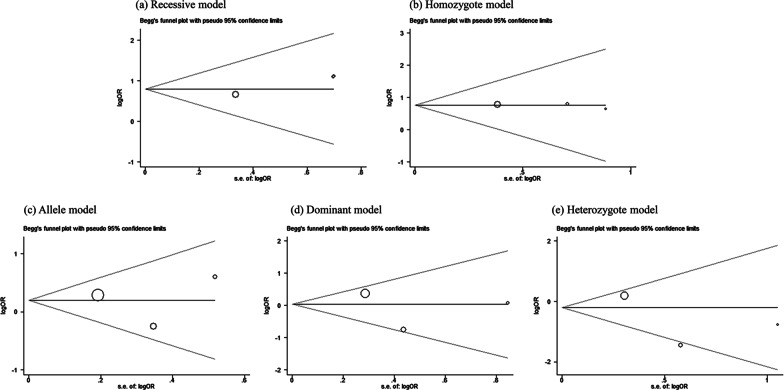
Begg’s test of publication bias in different models. **a** Recessive model; **b** Homozygote model; **c** Allele model; **d** Dominant model; **e** Heterozygote model

Publication bias indicated that meta-analysis often included published literatures tending to report statistically significant conclusions. The results of Begg’s test were listed in Table 2; Fig. 3. The *P* value of each study was more than 0.05, showing no publication bias in any genetic model.

## Discussion

Peri-implantitis affects the hard and soft tissues around the implant and causes irreversible bone loss, which is an important cause of implant failure [19]. Bone resorption is a typical feature of peri-implantitis. When the inflammation spreads to the alveolar bone, trap bone resorption occurs, due to the differentiated osteoclasts on the bone surface and in the bone marrow cavity [20]. Osteoclasts are highly differentiated multinucleated giant cells, mainly derived from monocyte/macrophage hematopoietic stem cell lineage, and play an important role in bone metabolism and bone resorption [21]. During osteoblast differentiation and maturation, the OPG acts as a regulatory hub and is a key regulatory cytokine [20]. OPG plays an important role in preventing resorption of alveolar bone [8]. It has been found that the proportion of OPG in gingival crevicular fluid of patients with periodontitis is down-regulated, and alveolar bone is severely damaged [22].

Single nucleotide mutations in genes can affect transcription and downstream protein production, and further affect the secretion of inflammatory factors and the regulation of immune response [23]. OPG single nucleotide polymorphism might affect the progression of peri-implantitis. It was reported that CC genotype of G1181C was related to the increased risk of peri-implantitis in a Chinese population [17]. But another study presented different results [11]. Therefore, a meta-analysis was needed to research the association between OPG single nucleotide polymorphism and peri-implantitis susceptibility.

There were a few studies on OPG gene polymorphism and peri-implantitis. After systematically retrieving the literature, three cross-sectional studies were finally included in the meta-analysis. Two studies found relationship between the CC genotype of OPG rs2073618 and increased risk of peri-implantitis [13, 17]. Another study found that OPG rs2073618 polymorphism was not associated with peri-implantitis [11]. This meta-analysis identified that the CC genotype of OPG rs2073618 in Recessive model and Homozygote model increased the risk of peri-implantitis. CC genotype of OPG G1181C had been proved to be related to lower bone density and lower serum OPG [24, 25]. Therefore, the CC genotype of OPG G1181C might result in the decreased level of OPG, which further leaded to weakened inhibition of osteoclast activation, and finally promoted the bone destruction in peri-implantitis. It had been reported that OPG level played an instructive role in terms of clinical diagnosis of peri-implantitis [26]. The results were consistent with this meta-analysis.

Besides rs2073618 polymorphism, there was another single nucleotide polymorphism T950C (rs2073617) of OPG, which was located in the in the promoter region [27]. T950C polymorphism indicated that Thymine at position 950 was replaced by Cytosine [28]. It was consistently reported in different studies that the T950C (rs2073617) of OPG was not associated with peri-implantitis [13, 17]. As a consequence, OPG rs2073617 polymorphism was not considered in this meta-analysis.

Receptor activator of nuclear factor k ligand (RANKL) belongs to tumor necrosis factor ligand super-family. RANKL combined with RANK to promote alveolar bone resorption, on the contrary of OPG [29]. OPG was the competitive receptor of RANKL. The RANK/RANKL/OPG pathway regulated the bone metabolism [30]. RANK/RANKL/OPG would be efficient biomarkers for making a distinction between peri-implantitis and healthy implants [29]. Nevertheless, some existing studies had indicated that the RANK and RANKL gene polymorphisms were found not to be related to the risk of peri-implantitis [11]. More related researches were needed.

Another systematic review [31] found that interferon, a proinflammatory biomarker, was shown to be more highly expressed in individuals with chronic periodontitis than it was in healthy patients. There was no conclusive evidence about how periodontal therapy affected the expression of interferon.

There were some limitations in this study. The number of eligible studies was small. Therefore, we could not conduct subgroup analysis to find the differences between different races. More studies of good quality were required.

## Conclusion

OPG rs2073618 polymorphism in Recessive model and Homozygote model was highly likely related to the risk of peri-implantitis. OPG might be the potential biomarker for identification of peri-implantitis, which is worthy of further exploration.

## Supplementary Information

**Additional file 1**. Table S1: Quality assessment and risk of bias of the included studies by NOS.

## Data Availability

The datasets used and/or analyzed during the current study are available from the corresponding author on reasonable request.
