# Supplementary material for: Association between Osteoprotegerin rs2073618 polymorphism and peri-implantitis susceptibility: a meta-analysis
Source: BMC Oral Health. 2022 Dec 12;22:598. doi: 10.1186/s12903-022-02657-6 (PMC9743567; doi:10.1186/s12903-022-02657-6)
Supplement: Supplementary file 1 — Additional file 1. Table S1: Quality assessment and risk of bias of the included studies by NOS. [file 12903_2022_2657_MOESM1_ESM.pdf]

Table S1: Quality assessment and risk of bias of the included studies by NOS.

Study	Assessment items	Score
Kadkhodazadeh et al. 2012	Selection	
	Is the case definition adequate?	★
	Representativeness of the cases	★
	Selection of Controls	
	Definition of Controls	★
	Comparability	
	Comparability of cases and controls on the basis of the design or analysis	★
	Exposure	
	Ascertainment of exposure	
	Same method of ascertainment for cases and controls	★
	Non-Response rate	★
	Total	7★
Silva et al. 2020	Selection	
	Is the case definition adequate?	★
	Representativeness of the cases	★
	Selection of Controls	
	Definition of Controls	★
	Comparability	
	Comparability of cases and controls on the basis of the design or analysis	★★
	Exposure	
	Ascertainment of exposure	★
	Same method of ascertainment for cases and controls	★
	Non-Response rate	★

	Total	8★
Zhou et al. 2016	Selection	
	Is the case definition adequate?	★
	Representativeness of the cases	★
	Selection of Controls	
	Definition of Controls	★
	Comparability	
	Comparability of cases and controls on the basis of the design or analysis	★
	Exposure	
	Ascertainment of exposure	★
	Same method of ascertainment for cases and controls	★
	Non-Response rate	★
	Total	7★
